# 1425. Revisiting β-Lactams for Treatment of Uncomplicated Urinary Tract Infections: Evaluation of Twice-daily Cephalexin for Empiric Treatment of UTIs

**DOI:** 10.1093/ofid/ofab466.1617

**Published:** 2021-12-04

**Authors:** Molly Benning, Dominic Acosta, Preeyaporn Sarangarm, Carla Walraven

**Affiliations:** University of New Mexico Hospitals, Albuquerque, New Mexico

## Abstract

**Background:**

Current IDSA guidelines for the treatment of UTIs discourage oral β-lactams based on lack of adequately powered studies to assess efficacy compared to fluoroquinolones or TMP-SMX. However, increasing *E. coli* and *Klebsiella spp.* resistance to first-line antibiotics has necessitated the need for alternative agents.

**Methods:**

This was a single-center retrospective chart review of adult patients discharged from the University of New Mexico ED with twice-daily cephalexin for the treatment of uncomplicated UTIs from January 1, 2019 to December 31, 2019. Patients were excluded if < 18 years of age, received ≥ 10 days of cephalexin, received antibiotics for other indications, received antibiotics within 60 days prior to ED visit, or had structural abnormalities. The primary outcome of this study was the proportion of patients with clinical success 30 days after discharge from the ED. Patients not meeting criteria for clinical failure were classified as clinical success. Clinical failure was defined as return of patient within 30 days due to non-resolving or worsening UTI symptoms or change in antibiotic therapy after discharge based on urine culture and susceptibilities.

**Results:**

A total of 264 patients were included for evaluation. The average age was 56.0 ± 20.2 years and 82.6% were female. Patients received an average 5.6 ± 0.9 days of antibiotic therapy including IV therapy. Of the 264 patients included for evaluation, 81.1% met criteria for clinical success. Of the patients with clinical failure, 29 (13.6%) required a change in antibiotics based on cultures and sensitivities, 17 (6.4%) returned for non-resolving or worsening symptoms, and 4 (1.5%) required both a change in antibiotics and returned for non-resolving or worsening symptoms.

**Conclusion:**

Short courses of twice-daily cephalexin appear to be safe and effective for empiric treatment of uncomplicated UTIs. Adding β -lactams back to the antibiotic armamentarium for UTI treatment may delay the development of resistance to non- β -lactam antibiotics, ensuring their future utility.

**Disclosures:**

**All Authors**: No reported disclosures

